# Effects of ondansetron exposure during ICU stay on outcomes of critically ill patients with sepsis: a cohort study

**DOI:** 10.3389/fcimb.2023.1256382

**Published:** 2023-12-21

**Authors:** Boshen Yang, Kaifan Niu, Yuankang Zhu, Xinjie Zheng, Taixi Li, Zhixiang Wang, Xian Jin, Xia Lu, Haifeng Qiang, Chengxing Shen

**Affiliations:** ^1^ Department of Cardiology, Shanghai Sixth People’s Hospital Affiliated to Shanghai Jiao Tong University School of Medicine, Shanghai, China; ^2^ Department of Gerontology, Xinhua Hospital Affiliated to Shanghai Jiaotong University School of Medicine, Shanghai, China; ^3^ Department of Cardiac Surgery, Xiamen University Affiliated Cardiovascular Hospital, Xiamen, China; ^4^ International Institutes of Medicine, Zhejiang University School of Medicine, Yiwu, China

**Keywords:** sepsis, ondansetron, treatment, immune response, prognosis

## Abstract

**Background:**

Sepsis is a life-threatening disease with high morbidity and mortality, characterized by an inadequate systemic immune response to an initial stimulus. Whether the use of ondansetron (OND) during intensive care unit (ICU) stay is associated with the prognosis of sepsis patients remains unclear.

**Methods:**

Critically ill patients with sepsis were extracted from the Medical Information Mart for Intensive Care IV (MIMIC-IV) database. Multivariate logistic regression and Cox regression analyses were used to explore the association between OND use and clinical outcomes after adjusting for confounders. Kaplan-Meier survival curve was used for survival analysis. Propensity score matching (PSM) and subgroup analysis were performed to further confirm the results.

**Results:**

The OND-medication group showed reduced in-hospital mortality, 28-day and 90-day mortalities. The OR for in-hospital mortality was 0.80 (0.64-0.99) and HRs for 28-day mortality and 90-day mortality were 0.77 (0.64-0.92) and 0.83 (0.70-0.98), respectively. After PSM, the clinical outcomes remained consistent. In-hospital mortality was lower in the OND-medication group (28.1% *vs*. 35.8%, P= 0.044), as well as 28-day mortality (23.4% *vs*. 32.1%, P=0.022) and 90-day mortality (27.4% *vs*. 35.8%, P=0.035). The protective effect of OND in sepsis patients was relatively robust, independent of age, septic shock, vasopressin and mechanical ventilation. Additionally, the OND users had longer lengths of stay in ICU (6.9(3.1-13.2) *vs*. 5.1(2.5-11.0), P = 0.026) while no statistical differences were found in lengths of stay in hospital (P = 0.333).

**Conclusion:**

OND exposure might be associated with lower in-hospital, 28-day, and 90-day mortality rates in critically ill patients with sepsis. This study indicated that OND might help improve the prognosis of patients with sepsis.

## Introduction

Sepsis is characterized by excessive systemic inflammatory and dysfunctional infection responses, which results in possible organ dysfunction and shock ([[NoAuthor]]; Kuipers et al., 2014; Bashar et al., 2020). Although progress has been made in the diagnosis and treatment of sepsis, it remains a life-threatening issue with high morbidity and mortality worldwide (Vincent et al., 2014; Angus et al., 2015; Rudd et al., 2020), especially for those in intensive care units (Baykara et al., 2018). Sepsis is one of the leading causes of death among critically ill patients admitted to intensive care unit (ICU) (Gaborit et al., 2021). Clinicians have tried multiple measures, including anti-infection treatment, organ protection, and fluid resuscitation to improve the survival rate of sepsis patients (Burke et al., 2019; Zhong et al., 2020). However, there is still no specific treatment for sepsis patients.

In recent years, accumulated evidence has shown that ondansetron (OND) might have broader pharmacological effects, especially in the anti-inflammation aspect. For instance, researchers found that OND attenuated pancreatic injury in the cerulein-induced acute pancreatitis model (Tsukamoto et al., 2017), the severity of dextran sulfate sodium salt-induced colitis (Utsumi et al., 2016) and targeted peritoneal macrophages as an anti-inflammatory agent (Maehara et al., 2015). These studies suggested that OND might have potential in treating inflammatory diseases. Some indicators reflecting the level of systemic inflammation, including neutrophil to lymphocyte ratio (NLR) and platelet to lymphocyte ratio (PLR), were highly correlated with mortality in septic patients (Kaushik et al., 2018; Shen et al., 2019; Huang et al., 2020). Interestingly, researchers found that OND pre-treatment decreased the mortality of ICU patients on mechanical ventilation, which could be explained by regulation of NLR (Zhou et al., 2022).

It has been reported that the use of OND may be associated with a potential prolongation of the cardiac QT interval (Charbit et al., 2008). However, it is important to note that when ondansetron is administered orally at therapeutic doses, the likelihood of it causing a clinically significant prolongation of the QT interval is very low, and the risk of inducing arrhythmias is even lower (Freedman et al., 2014). Furthermore, the use of OND during pregnancy has not been found to be significantly linked to an increased risk of adverse fetal outcomes, including conditions like cardiac malformations, oral clefts, or congenital malformations overall (Pasternak et al., 2013; Huybrechts et al., 2020). Therefore, it is appealing for us to investigate whether OND medication during ICU stay plays a protective role in septic patients or not.

In the present study, we aimed to explore the effects of OND exposure during ICU stay on clinical outcomes of septic patients, including in-hospital, 28-day, 90-day mortality rates and length of stay in hospital and ICU. Our research might provide a new treatment option for patients with sepsis in ICU to forecast and improve their prognosis.

## Materials and methods

### Data source

In the present study, we used a high-quality and large database, which was called the Medical Information Mart for Intensive Care IV (MIMIC-IV) database. MIMIC-IV database contains critically ill patients admitted to ICU at Beth Israel Deaconess Medical Center (BIDMC) between 2008-2019 (Johnson et al., 2021). All the clinical data, including demographics, hospital and ICU admission and discharge time, vital signs, laboratory data, medications and nursing records were recorded in this database. One of our partners passed the Protection of Human Research Participants Examination and was allowed to access the database. SQL (structured query language) was employed to extract data from the MIMIC-IV database.

### Study participants

All hospital admissions were obtained from the MIMIC-IV database, and patients with sepsis or septic shock were identified using the International Classification of Diseases (ICD)-9 codes, including 99591 and 99592. Patients who had no ICU stay records or were younger than 18 years old were excluded. For patients who had more than one hospital admission or ICU admission records, we only kept their first ICU experience in the first hospital admission for final analysis. Patients that had OND treatment records during ICU stay were included in OND-medication groups and those who did not were included in non- OND-medication group.

### Clinical data

Demographics, vital signs, comorbidities, laboratory data and treatment measures of each patient were extracted from the database. Demographics contained age, gender, and weight, while vital signs include systolic blood pressure (SBP), diastolic blood pressure (DBP), respiratory rate (RR), heart rate (HR) and temperature. As for comorbidities, cerebral diseases, atrial fibrillation (AF), chronic kidney disease (CKD), acute kidney injury (AKI), chronic heart failure (CHF), and septic shock were extracted of each patient for analysis. Laboratory data, including red blood cell (RBC), white blood cell (WBC), platelet, hemoglobin, creatinine, glucose, lactate, potassium and chloride, were summarized as baseline characteristics. Additionally, to reflect the disease severity of each patient, the first measurement of Sequential Organ Failure Assessment (SOFA) of each patient within 24 hours during ICU stay was included. Regarding treatment measures, mechanical ventilation, vasopressin and antibiotic uses were included. Vital signs and clinical indices were defined as the first measurement after entering ICU. Multiple imputation (MI) is a commonly used statistical technique for handling missing data (Johnson et al., 2021). Vital signs and clinical indices with missing values exceeding 30% were removed, and the remaining missing values were imputed using MI in Stata (version 14.0). With MI, several plausible values for a specific variable are imputed or filled in for each subject who has missing data for that variable.

### Clinical outcomes

The primary outcomes of this study were defined as all-cause in-hospital mortality, 28-day mortality, and 90-day mortality. The secondary outcomes were defined as lengths of stay (LOS) in ICU or in hospital. 28-day or 90-day mortalities were defined as patients who died within 28 days or 90 days after ICU admission.

### Statistical analysis

Data was summarized in tables and displayed according to their distributions and types of variables. Categorical variables were presented as numbers (percentages), which were tested by Chi- square or Fisher’s exact tests. Continuous variables were displayed as mean ± standard deviation or median (25-75 percentiles) and tested by student’s t-test or Wilcoxon rank-sum tests. Logistic or Cox regression analyses was used to explore the association between OND and in-hospital or 28-day, 90-day mortalities. Multivariate logistic or Cox regression analyses was further performed to avoid bias induced by confounders. Propensity score matching (PSM) is a highly used method for balancing potential influencing factors in research populations and evaluating the robustness of results. 1:1 PSM was also employed with no replacement via the nearest neighbor, and the caliper width was set at 0.02. All possible influencing factors were taken into account in the PSM cohorts.

All statistical analyses in this study were conducted using SPSS (version 23.0) or Stata (version 14.0). Survival analysis was performed using Stata (version 14.0) and a Log-rank test was used to evaluate it. P-Value lower than 0.05 was set for statistical significance in the present study.

## Results

### Baseline characteristics and clinical outcomes of the study participants

**Figure 1:**
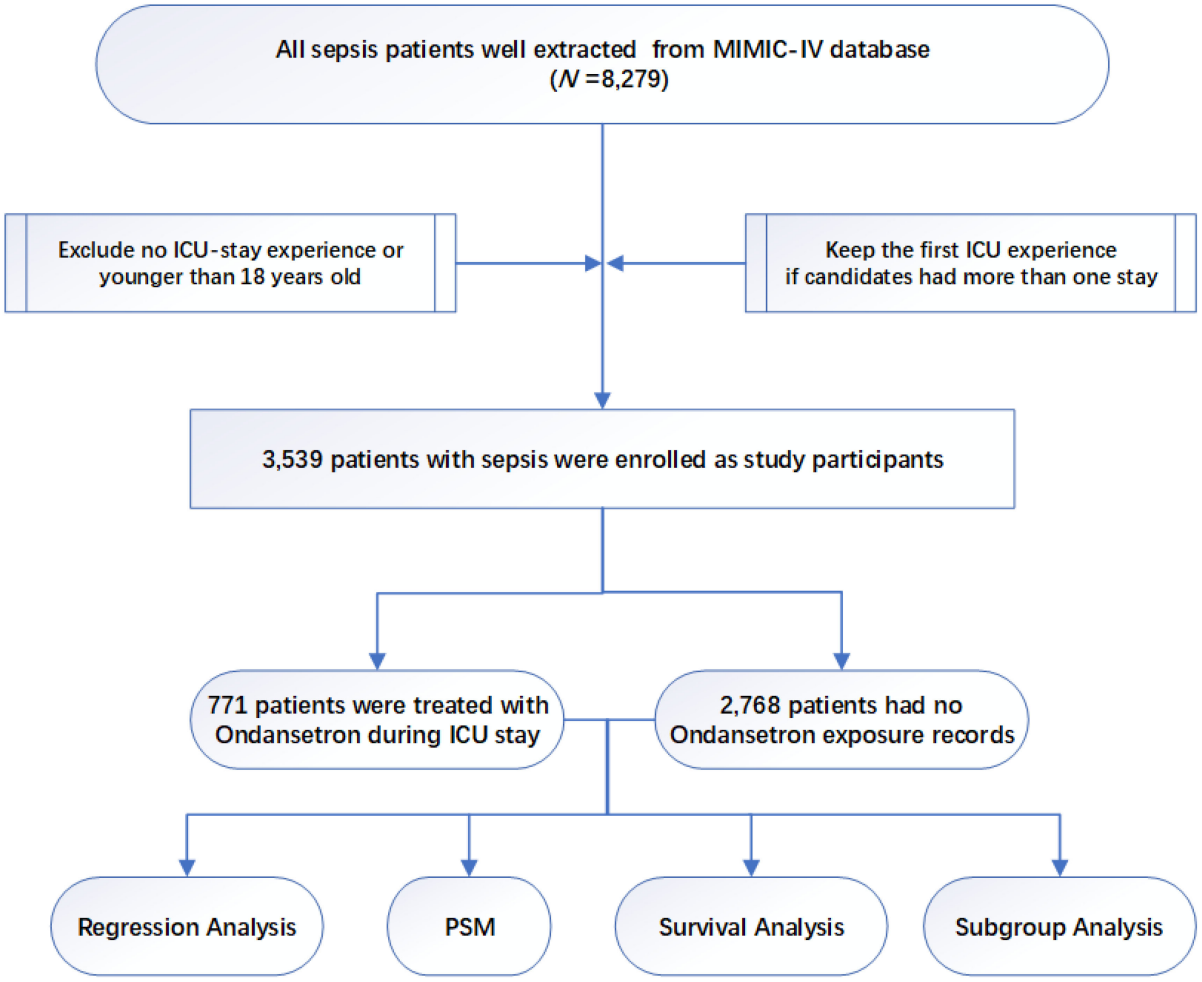
Study flowchart. PSM, propensity score matching. .

The study design is displayed as a flowchart in **Figure 1**. After selection based on the study design, a total of 3539 patients with sepsis were enrolled in the final cohort. Among patients, 771 of them had OND exposure records during ICU stay while 2768 of them did not. Baseline characteristics, including demographic data, vital signs, comorbidities, laboratory data, and treatment measures were extracted from the database and are displayed in **Table 1**. The data showed that patients in OND-medication group were younger and had a higher proportion of males. RR and DBP were close between the two groups though differences were significant. No statistical differences were found in the ethnic composition of the two groups. A higher incidence of AKI was observed in sepsis patients who were not treated with OND. Regarding laboratory data, no significant differences were observed in WBC, RBC, hemoglobin, creatinine, and glucose. Lactate was lower in patients treated with OND. Potassium and chloride were close between the two groups while significant differences were shown. Treatment measures between the two groups, such as vasopressin, antibiotic use and mechanical ventilation, were similar.

**Table 1:** Baseline characteristics of study participants.

	All patients (n=3539)	Non-ondansetron medication group (n=2768)	ondansetron medication group (n=771)	P-value
Demographic data				
Age (years)	68 (56-80)	69 (57-81)	64 (52-78)	<0.001
Male (n (%))	1573 (44.4)	1162 (42.0)	411 (53.3)	<0.001
Weight (Kg)	78 (65-95)	78 (66-94)	78 (64-95)	0.735
Race White Black Asian Hispanic Other	2399 (67.8) 348 (9.8) 139 (3.9) 131 (3.7) 522 (14.7)	1864 (67.3) 286 (10.3) 105 (3.8) 94 (3.4) 419 (15.1)	535 (69.3) 62 (8.0) 34 (4.4) 37 (4.8) 103 (13.4)	0.281 0.059 0.068 0.436 0.218
Vital signs				
RR (/min)	21 (18-24)	21 (18-24)	21 (18-23)	0.006
SBP (mmHg)	113 (98-130)	113 (98-130)	111 (98-127)	0.133
DBP (mmHg)	57 (49-67)	57 (49-67)	58 (49-68)	0.016
Temperature (°C)	36.9 (36.6-37.3)	36.9 (36.6-37.3)	36.9 (36.6-37.4)	0.046
HR (/min)	91 (80-104)	91 (80-103)	92 (80-104)	0.128
Comorbidities				
Cerebral diseases (n (%))	399 (11.3)	319 (11.5)	80 (10.4)	0.403
AHF (n (%))	611 (17.3)	496 (17.9)	115 (14.9)	0.052
AF (n (%))	192 (5.4)	157 (5.7)	35 (4.5)	0.243
CKD (n (%))	858 (24.2)	675 (24.4)	183 (23.7)	0.740
AKI (n (%))	2257 (63.8)	1807 (65.3)	450 (58.4)	0.001
CHF (n (%))	749 (21.2)	585 (21.1)	164 (21.3)	>0.9
Septic shock (n (%))	2043 (57.7)	1604 (57.9)	439 (56.9)	0.621
Clinical indices				
RBC (m/uL)	3.3 (2.9-3.8)	3.3 (2.9-3.8)	3.3 (2.8-3.8)	0.437
WBC (K/uL)	10.3 (6.9-15.5)	10.3 (7.0-15.4)	10.4 (6.6-15.7)	0.358
Platelet (K/uL)	191 (123-282)	191 (124-287)	191 (117-264)	0.028
Hemoglobin	9.9 (8.7-11.2)	9.8 (8.6-11.3)	9.9 (8.7-11.3)	0.863
Creatinine (mg/dL)	1.1 (0.7-2.0)	1.2 (0.7-2.0)	1.0 (0.7-1.9)	0.585
Glucose (mmol/L)	118 (96-154)	119 (96-155)	116 (95-152)	0.434
Lactate (mg/dL)	1.7 (1.2-2.8)	1.7 (1.2-2.8)	1.6 (1.2-2.8)	0.003
Potassium (mmol/L)	4.0 (3.7-4.4)	4.0 (3.7-4.5)	4.0 (3.7-4.4)	0.075
Chloride (mmol/L)	104 (100-108)	104 (100-108)	104 (100-108)	0.035
SOFA	7 (4-11)	7 (4-11)	6 (4-10)	<0.001
Treatment measures				
Vasopressin (n (%))	837 (23.7)	649 (23.4)	188 (24.4)	0.736
Antibiotic (n (%))	3468 (98.0)	2710 (97.9)	758 (98.3)	0.562
Mechanical Ventilation (n (%))	2991 (84.5)	2350 (84.9)	641 (83.1)	0.237

RR, respiratory rate; HR, heart rate; SBP, systolic blood pressure; DBP, diastolic blood pressure; AF, atrial fibrillation; AHF, acute heart failure; CKD, chronic kidney disease; AKI, acute kidney injury; CHF, chronic heart failure; RBC, red blood cell; WBC, white blood cell; SOFA, Sequential Organ Failure Assessment.

As shown in **Table 2**, the results showed that OND exposure during ICU stay significantly reduced the 28-day (20.2% *vs*. 27.5%) and 90-day (24.6% *vs*. 30.9%) mortalities in patients with sepsis as well as declined in-hospital mortality (23.1% *vs*. 29.2%). Interestingly, the OND medication group had significantly longer lengths of stay in ICU [3.5(1.9-8.5) *vs*. 2.9(1.6-6.8)] while no difference was observed in hospital LOS between the two groups (P=0.388).

**Table 2:** Clinical outcomes of study participants.

	All patients (n=3539)	Non-ondansetron medication group (n=2768)	ondansetron medication group (n=771)	P-value
Primary outcomes				
In-hospital mortality (n (%))	987 (27.9)	809 (29.2)	178 (23.1)	0.001
28-mortality (n (%))	917 (25.9)	761 (27.5)	156 (20.2)	<0.001
90-mortality (n (%))	1044 (29.5)	854 (30.9)	190 (24.6)	0.001
Secondary outcomes				
ICU LOS (days)	3.0 (1.6-7.2)	2.9 (1.6-6.8)	3.5 (1.9-8.5)	<0.001
Hospital LOS (days)	9.9 (5.5-17.9)	9.9 (5.5-17.7)	9.9 (5.7-19.0)	0.388

ICU, intensive care unit; LOS, length of stay.

### Survival analysis

**Figure 2:**
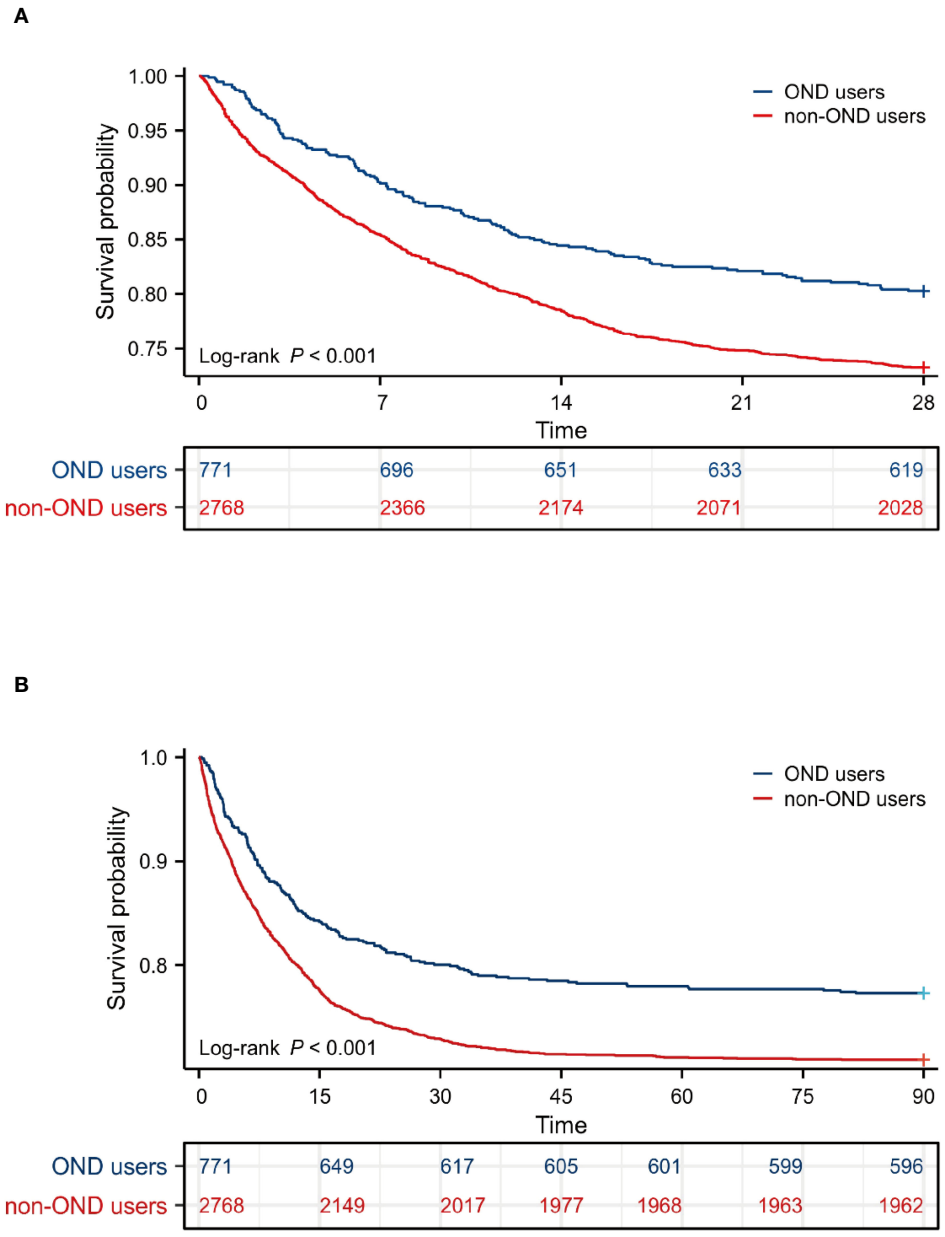
Survival analysis. **(A)** Kaplan-Meier survival curve of the two groups within 28 days. **(B)** Kaplan-Meier survival curve of the two groups within 90 days.

Kaplan-Meier survival curves were drawn to intuitively reflect the mortality risk of the two groups of patients. The survival period of each patient was extracted from the database. As shown in **Figure 2**, patients with sepsis treated with OND had a higher probability of survival within 28 days and 90 days. The P-values of log-Rank test were all lower than 0.05 for two curves.

### Association between OND exposure and mortality risk using multivariate regression analysis

**Figure 3:**
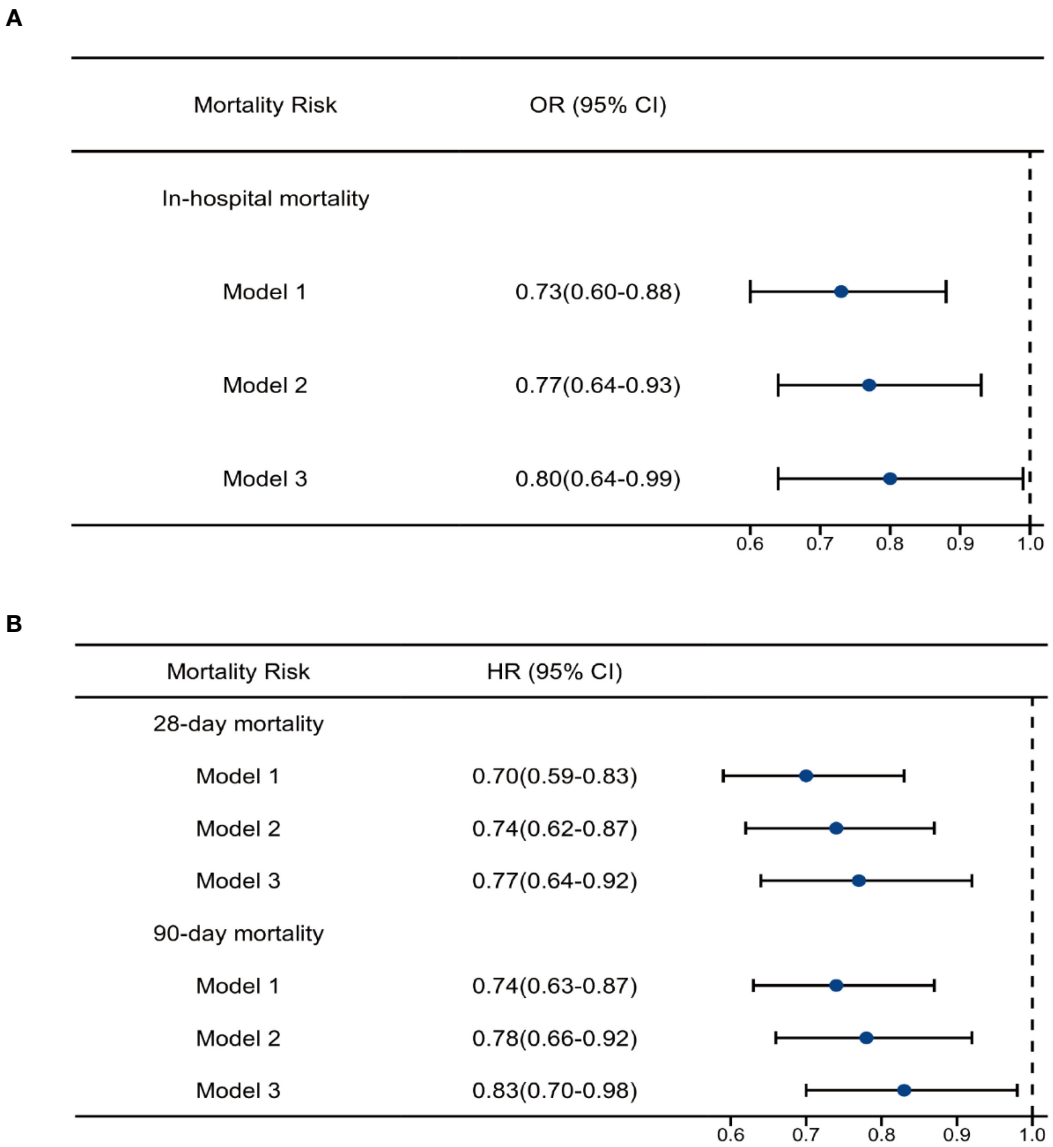
Regression analysis. **(A)**Multivariate logistic analysis of ondansetron use on in-hospital mortality **(B)** Multivariate Cox analysis of ondansetron use on 28-day and 90-day mortality. OND, ondansetron; HR, hazard ratio; OR, odds ratio.

Multivariate logistic regression analysis was used to explore the association between OND treatment and all-cause in-hospital mortality, and multivariate Cox regression was used for 28-day and 90-day mortalities. As shown in **Table 3**; **Figure 3**, in the unadjusted model 1, HRs for 28-day and 90-day mortalities were 0.70(0.59-0.83) and 0.74(0.63-0.87), respectively. The OR for in-hospital mortality was 0.73 (0.60-0.88). After adjusting for age, gender and weight, the trend remained consistent in model 2. Furthermore, we adjusted for age, gender, weight, heart rate, respiratory rate, temperature, systolic blood pressure, cerebral disease, AHF, CHF, CKD, AKI, white blood cell, red blood cell, lactate, glucose, creatinine, platelet, hemoglobin, septic shock, antibiotic use, mechanical ventilation and vasopressin in model 3 and the results were robust. HRs for 28-day and 90-day mortality were 0.77 (0.64-0.92) and 0.83 (0.70-0.98) while OR for in-hospital mortality was 0.80 (0.64-0.99). Taken together, these results revealed that OND exposure might play a protective role in critically ill patients with sepsis.

**Table 3:** Association between OND treatment and clinical outcomes using multivariate regression analysis.

	HR for 28-day mortality	HR for 90-day mortality	OR for in-hospital mortality
Model 1	0.70 (0.59-0.83)	0.74 (0.63-0.87)	0.73 (0.60-0.88)
Model 2	0.74 (0.62-0.87)	0.78 (0.66-0.92)	0.77 (0.64-0.93)
Model 3	0.77 (0.64-0.92)	0.83 (0.70-0.98)	0.80 (0.64-0.99)

**Model 1** was unadjusted.

**Model 2** was adjusted by age, gender and weight.

**Model 3** was adjusted by age, gender, weight, heart rate, respiratory rate, temperature, systolic blood pressure, cerebral disease, AHF, CHF, CKD, AKI, white blood cell, red blood cell, lactate, glucose, creatinine, platelet, hemoglobin, septic shock, antibiotic use, mechanical ventilation, vasopressin.

OND, ondansetron; HR, hazard ratio; OR, odds ratio.

### Baseline characteristics and clinical outcomes after propensity score matching

To further avoid the effects of confounders in this cohort, we employed 1:1 matched PSM analysis to further confirm the results. A total of 598 patients were enrolled after PSM. Among them, 299 patients were treated with OND during ICU stay while 299 patients not. As shown in **Table 4**, all baseline characteristics between the two groups were similar with P-value higher than 0.05. Interestingly, the clinical outcomes remained consistent after PSM. As displayed in **Table 5**, in-hospital mortality (35.8% *vs*. 28.1%, p= 0.044), as well as 28-day mortality (32.1% *vs*. 23.4%, p= 0.022) and 90-day mortality (35.8% *vs*. 27.4%, p=0.035), were all lower in OND-medication group compared with those not. The OND users had longer LOS in ICU [5.1(2.5-11.0) *vs*. 6.9(3.1-13.2), p= 0.026] while no statistical differences were found in LOS of hospital [14.3(8.5-22.7) *vs*. 15.5(8.5-2.3), P = 0.333]. These results further demonstrated that OND exposure during ICU stay might be associated with improved prognosis in critically ill patients with sepsis.

**Table 4:** Baseline characteristics of study participants after propensity score matching.

	All patients (n=598)	Non-ondansetron medication (n=299)	ondansetron medication (n=299)	P-value
Demographic data				
Age (years)	64 (52-76)	64 (52-77)	65 (53-76)	0.872
Male (n (%))	295 (49.3)	147 (49.2)	148 (49.5)	>0.9
Weight	80 (68-100)	80 (68-99)	83 (67-100)	0.821
Race				
White	401 (67.1)	199 (66.6)	202 (67.6)	0.794
Black	50 (8.4)	27 (9.0)	23 (7.7)	0.555
Asian	0 (0)	0 (0)	0 (0)	/
Hispanic	22 (3.7)	10 (3.3)	12 (4.0)	0.664
Other	125 (20.9)	63 (21.1)	62 (20.7)	0.920
Vital signs				
RR (/min)	20 (18-24)	20 (18-24)	21 (18-24)	0.740
SBP (mmHg)	111 (99-128)	112 (98-129)	110 (99-126)	0.819
DBP (mmHg)	57 (49-67)	57 (49-67)	57 (50-67)	0.740
Temperature (°C)	36.9 (36.6-37.4)	36.9 (36.6-37.4)	36.9 (36.6-37.4)	0.156
HR (/min)	91 (79-104)	91 (77-105)	91 (79-102)	0.895
Comorbidities				
Cerebral disease (n (%))	77 (12.9)	35 (11.7)	42 (14.0)	0.464
AHF (n (%))	121 (20.2)	64 (21.4)	57 (19.1)	0.541
AF (n (%))	32 (5.4)	14 (4.7)	18 (6.0)	0.586
CKD (n (%))	186 (31.1)	97 (32.4)	89 (29.8)	0.536
AKI (n (%))	407 (68.1)	204 (68.2)	203 (67.9)	>0.9
CHF (n (%))	176 (29.4)	91 (30.4)	85 (28.4)	0.654
Septic shock (n (%))	381 (63.7)	191 (63.9)	190 (63.5)	>0.9
Cinical indices				
RBC (m/uL)	3.1 (2.8-3.6)	3.0 (2.8-3.6)	3.2 (2.8-3.6)	0.603
WBC (K/uL)	10.8 (7.1-16.2)	10.4 (7.0-15.9)	11.4 (7.3-16.8)	0.166
Platelet (K/uL)	183 (118-259)	179 (109-261)	187 (123-258)	0.549
Hemoglobin	9.5 (8.4-10.8)	9.5 (8.3-10.8)	9.5 (8.4-10.9)	0.909
Creatinine (mg/dL)	1.2 (0.7-2.1)	1.2 (0.8-2.2)	1.2 (0.7-1.9)	0.504
Glucose (mmol/L)	122 (100-154)	123 (100-154)	122 (100-155)	0.526
Lactate (mg/dL)	1.6 (1.1-2.5)	1.6 (1.1-2.5)	1.6 (1.1-2.5)	0.577
Potassium (mmol/L)	4.1 (3.7-4.5)	4.1 (3.7-4.5)	4.0 (3.7-4.5)	0.579
Chloride (mmol/L)	104 (99-108)	104 (100-108)	103 (99-108)	0.467
SOFA	9 (5-12)	8 (6-12)	9 (5-12)	0.859
Clinical measures				
Vasopressin (n (%))	239 (40.0)	116 (38.8)	123 (41.1)	0.616
Antibiotic (n (%))	598 (100)	299 (100)	299 (100)	>0.9
Mechanical Ventilation (n (%))	570 (95.3)	286 (95.7)	284 (95.0)	0.847

RR, respiratory rate; HR, heart rate; SBP, systolic blood pressure; DBP, diastolic blood pressure; AF, atrial fibrillation; AHF, acute heart failure; CKD, chronic kidney disease; AKI, acute kidney injury; CHF, chronic heart failure; RBC, red blood cell; WBC, white blood cell; SOFA, Sequential Organ Failure Assessment.

**Table 5:** Clinical outcomes of study participants after propensity score matching.

	All patients (n=598)	Non-ondansetron medication (n=299)	ondansetron medication (n=299)	P-value
Primary outcomes				
In-hospital mortality (n (%))	191 (31.9)	107 (35.8)	84 (28.1)	0.044
28-mortality (n (%))	166 (27.8)	96 (32.1)	70 (23.4)	0.022
90-mortality (n (%))	189 (31.6)	107 (35.8)	82 (27.4)	0.035
Secondary outcomes				
ICU LOS (days)	6.0 (2.8-12.5)	5.1 (2.5-11.0)	6.9 (3.1-13.2)	0.026
Hospital LOS (days)	14.8 (8.5-24.7)	14.3 (8.5-22.7)	15.5 (8.5-25.3)	0.333

ICU, intensive care unit; LOS, length of stay.

**Figure 4:**
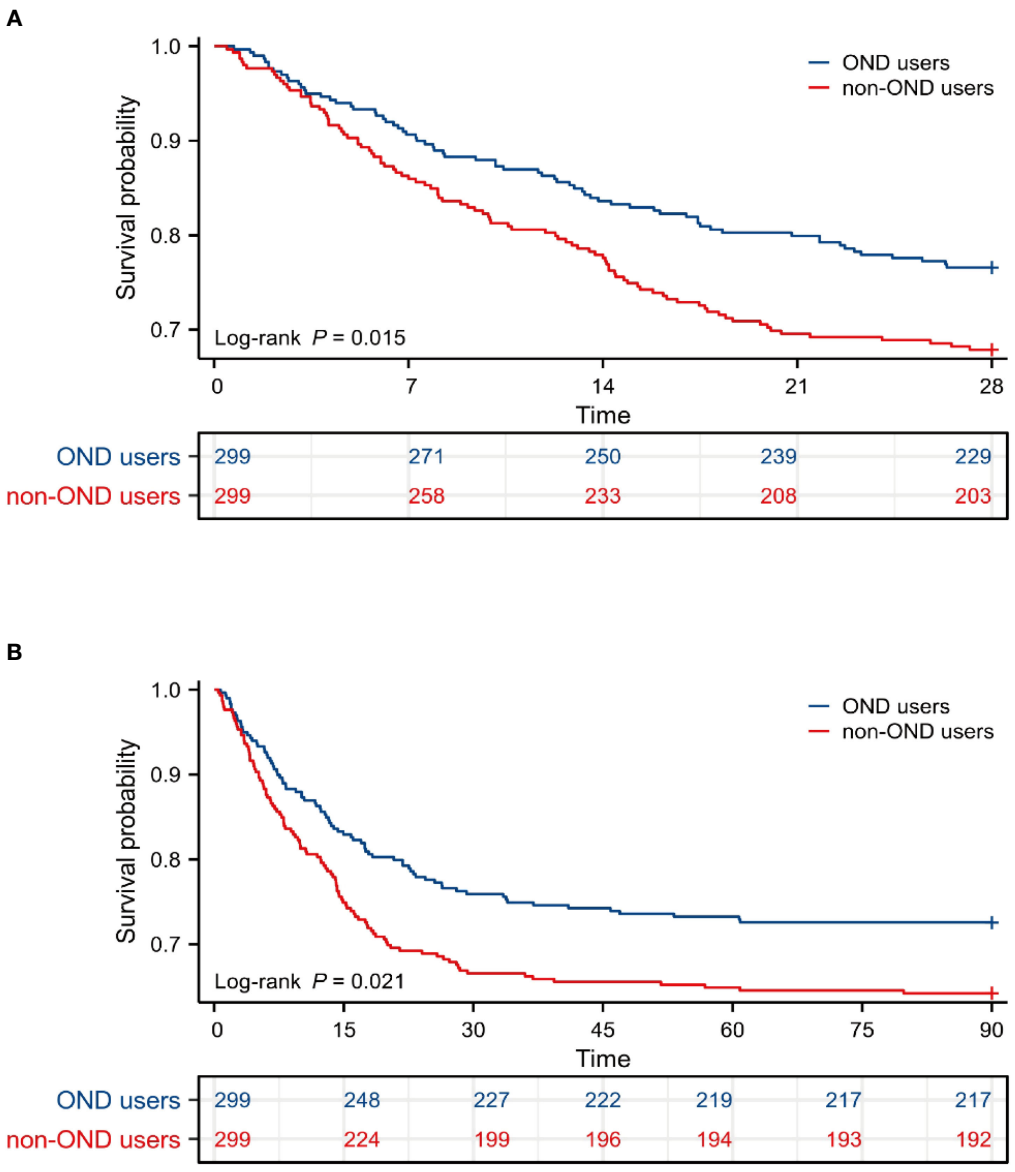
Survival analysis after PSM. **(A)** Kaplan-Meier survival curve of the two groups within 28 days after PSM. **(B)** Kaplan-Meier survival curve of the two groups within 90 days after PSM. PSM, propensity score matching.

As displayed in **Figure 4**, Kaplan-Meier survival curves showed OND-users had a higher survival probability within 28 days and 90 days after PSM. P-values for log-rank tests were lower than 0.05.

### Subgroup analysis

**Figure 5:**
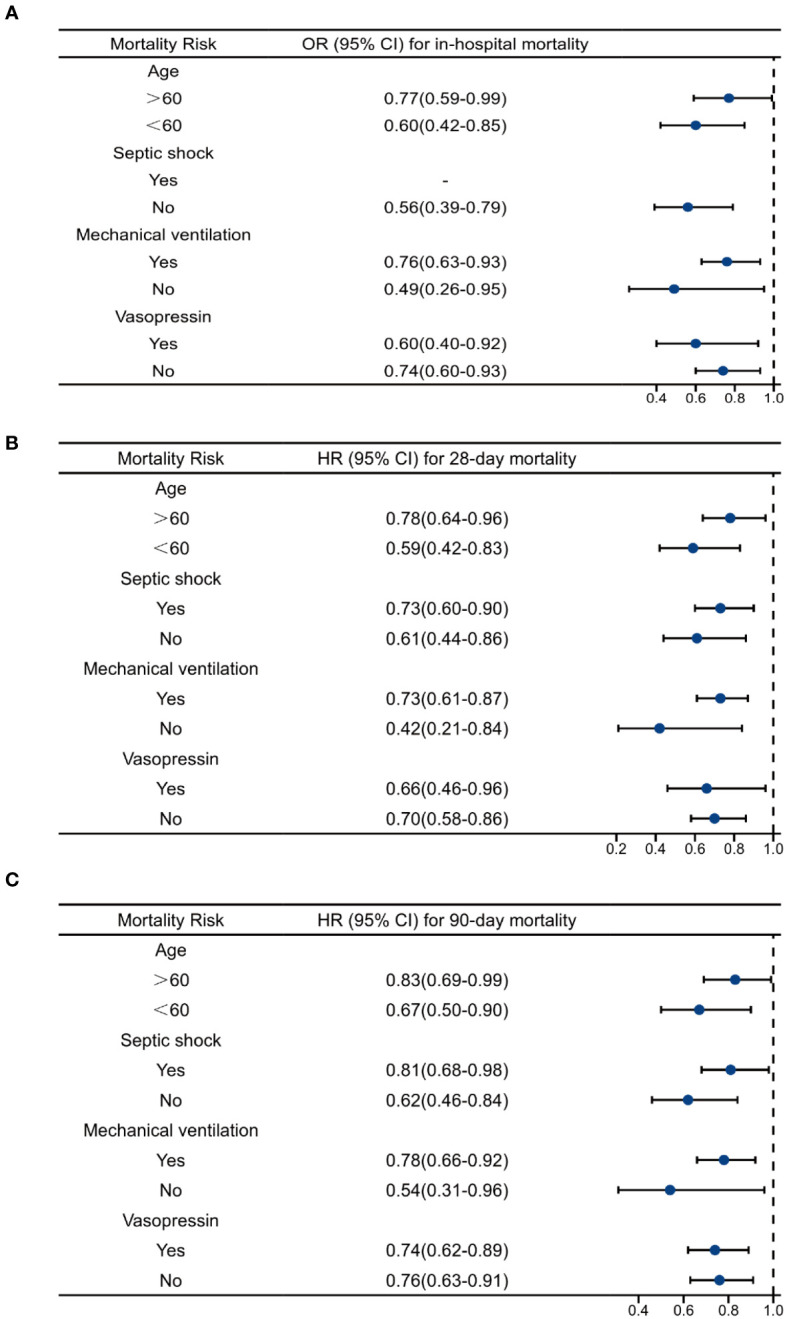
Multivariate regression analysis of ondansetron use on clinical outcomes in different subgroups. **(A)**Multivariate logistics analysis of ondansetron use on in-hospital mortality. **(B)** Multivariate Cox analysis of ondansetron use on 28-day mortality. **(C)** Multivariate Cox analysis of ondansetron use on 90-day mortality. OND, ondansetron; HR, hazard ratio; OR, odds ratio.

We performed subgroup analysis according to whether the patient developed septic shock and whether mechanical ventilation and vasoactive drugs were used during ICU stay. As shown in **Table 6**; **Figure 5**, the use of mechanical ventilation and vasopressin did not alter the protective effects of OND in sepsis patients. In patients with septic shock, OND treatment was associated with lower mortality risk for 28-day mortality [0.73(0.60-0.90)] and 90-day mortality 0.81(0.68-0.98). Interestingly, the protective effect of OND for in-hospital mortality disappeared in patients with septic shock. Taken together, these results showed that effects of OND on clinical outcomes in sepsis patients were relatively robust while might be influenced by different disease severity. Additionally, OND still had a protective effect in elderly patients (Age>60).

**Table 6:** Association between OND treatment and clinical outcomes stratified by septic shock, mechanical ventilation, vasopressin and age.

	HR for 28-day mortality	HR for 90-day mortality	OR for in-hospital mortality
Septic shock			
Yes	0.73 (0.60-0.90)	0.81 (0.68-0.98)	–
No	0.61 (0.44-0.86)	0.62 (0.46-0.84)	0.56 (0.39-0.79)
Mechanical Ventilation			
Yes No	0.73 (0.61-0.87) 0.42 (0.21-0.84)	0.78 (0.66-0.92) 0.54 (0.31-0.96)	0.76 (0.63-0.93) 0.49 (0.26-0.95)
Vasopressin			
Yes No	0.66 (0.46-0.96) 0.70 (0.58-0.86)	0.74 (0.62-0.89) 0.76 (0.63-0.91)	0.60 (0.40-0.92) 0.74 (0.60-0.93)
Age			
>60	0.78 (0.64-0.96)	0.83 (0.69-0.99)	0.77 (0.59-0.99)
<60	0.59 (0.42-0.83)	0.67 (0.50-0.90)	0.60 (0.42-0.85)

OND, ondansetron; HR, hazard ratio; OR, odds ratio.

## Discussion

In the present study, we explored the possible association between OND use during ICU stay with sepsis and the clinical outcomes in critically ill patients. We found that the use of OND might relate to lower in-hospital mortality of sepsis patients and improved long-term prognosis, evident by lower 28-day and 90-day mortalities. Combining with multivariate logistic or Cox regression analyses and propensity score matching, the potential confounders were balanced for a more robust conclusion. Taken together, this study indicated that OND exposure could be a protective factor in critically ill patients with sepsis. Therefore, our study revealed the unrecognized and promising role of OND in sepsis patients, which might be a novel potential treatment strategy for these high-risk patients.

Sepsis or septic shock was defined as a life-threatening organ dysfunction caused by a dysregulated host response to infection (Singer et al., 2016), characterized by an inadequate systemic immune response to an initial stimulus. There is no doubt that sepsis is a major health issue worldwide, and it is estimated that 5.3 million people die of this disease every year (Vincent et al., 2006; Kaukonen et al., 2014; Fleischmann et al., 2016). To make matters worse, accumulated evidence revealed that the number of sepsis patients is rising rapidly (Martin et al., 2003; Lagu et al., 2012; Shankar-Hari et al., 2017). Hence, it is essential for us to diagnose and treat sepsis patients at an early stage.

In current medical practice, the treatment of sepsis is still limited, which can be summarized into three aspects, including infection control, hemodynamic management and modulation of the host response (Vincent, 2022). Antibiotics were necessary for nearly every patient at an early stage even when no particular microorganism was isolated from blood (Sakr et al., 2018; Lakbar et al., 2022). Regarding hemodynamic management, fluid administration and vasopressin, such as noradrenaline or dobutamine, were also required in some situations for an initial mean arterial pressure (MAP) of 65 mmHg was recommended (Evans et al., 2021).Immunomodulatory therapy is gradually gaining traction in sepsis, because early and over-activation of the immune inflammatory response is a major cause of infectious death (Liu et al., 2022). Low-dose of GM-CSF could improve oxygenation index in septic patients with respiratory dysfunction but had no positive effect on 30-day mortality (Orozco et al., 2006). Similarly, a phase-II clinical study showed that granulocyte-macrophage colony-stimulating factor therapy decreased antibiotics treatment time and infectious complications but didn’t improve the in-hospital mortality rate of sepsis patients (Presneill et al., 2002). Combination therapy of ulinastatin and Tα1 reduce 28d and 90d mortality rates and organ damages in sepsis patients while the effect of the medication alone use was not satisfactory (Han et al., 2015; Liu et al., 2017). This evidence suggest that immune regulation might be a potential treatment option for sepsis patients.

OND, as a 5-HTR antagonist, had a binding ability to 5-HT_3_R 5-HT_1B_R, 5- HT_1C_R, α-adrenergic receptor and μ-opioid receptor (Kovac, 2016). In recent years, studies found that 5-HT_3_R antagonists might have pharmacological effects in many diseases, particularly their anti-inflammatory effect. For instance, tropisetron showed protective effect by mediating 5-HT_3_ receptors (Motavallian et al., 2013) in inflammatory bowel disease and granisetron could ameliorate acetic acid-induced colitis (Fakhfouri et al., 2010). Local injection of tropisetron potently relieved inflammation and pain in arthritis, osteoarthritis and tendinopathies (Stratz and Müller, 2000; Stratz et al., 2002). The cytokine storm caused by excessive inflammatory activity is a key issue during the progression of sepsis (Karbian et al., 2020). Researchers have discovered that intervention with a 5-HT3 receptor antagonist can help control the overproduction of pro-inflammatory cytokines involved in the pathogenesis of severe sepsis or septic shock, and reduce serum levels of noradrenaline in sepsis models (Stratz et al., 2002; Setoguchi et al., 2011). Oxidative stress resulting from the release of reactive oxygen species (ROS) contributes to mitochondrial dysfunction, induces cell apoptosis, and worsens the prognosis of sepsis patients (Huet et al., 2011). Therefore, antioxidant therapy is considered a potential strategy to improve sepsis outcomes. An animal study reported that the use of OND could alleviate oxidative stress in rats with cystitis (Zirak et al., 2020). Similarly, OND was found to maintain the pro-oxidant/antioxidant balance in the brain by increasing GSH concentrations and inhibiting MDA in a mouse model (Gupta et al., 2014). Collectively, these findings suggest that OND might mitigate sepsis-related injuries through its anti-inflammatory and antioxidant properties, although the specific molecular mechanisms still require further investigation.

In current clinical practice, ondansetron (OND) is commonly used to prevent and treat nausea and vomiting in critical care settings. However, emerging evidence suggests that OND may also have a role in improving the prognosis of critically ill patients. This highlights the potential for OND to provide benefits beyond its established use for managing nausea and vomiting. As previously reported, OND exposure was associated with lower 90-day mortality for cardiac surgical patients and postoperative AKI (Xiong and Xiong, 2022). Meanwhile, Gray et al. also considered the use of OND is friendly with kidney and was associated with a significance decrease in 90-day mortality in AKI patients compared with other anti-emetics (Gray et al., 2022). They were consistent with our results and indicated that OND might be a potential clinical treatment for critically ill patients to improve their outcomes. Researchers have also discovered that the 5-HT3 receptor antagonist could reduce the mortality of septic mice (Gong et al., 2019). This suggests that drugs targeting the 5-HT3 receptor may have a positive impact on the survival of mice with sepsis. Additionally, the OND medication was associated with longer lengths of stay in ICU in this study, which might be due to the shorter survival time of non-OND user, thereby shortening their ICU LOS. Therefore, it is necessary to conduct randomized controlled trials in the future to confirm whether OND should be included as a routine medication for septic patients. Such trials will help determine the effectiveness and safety of using OND in the management of sepsis and provide more concrete evidence to guide clinical practice.

There are several limitations in the present study. First, this is a single-center retrospective study, although we have adopted various methods to exclude potential migrations, it may still exist. Secondly, we have not subdivided the dosage and initiated time of OND, as well as the duration of treatment time, although they were not specified, they may also act as a factor affecting the clinical outcomes of patients. Furthermore, it’s worth noting that the septic patients in this study were identified solely based on ICD-9 coding, without the use of any other clinical criteria, potentially limiting the generalizability of the study’s findings. Additionally, some clinical indices with more than 30% missing values, including neutrophil and lymphocyte counts, were excluded in this study. However, it cannot be denied that these excluded variables may have had an impact on the study’s outcomes. Despite the extensive adjustment for numerous potential confounding factors, it’s important to acknowledge that there may still be unaccounted variables that could have influenced the results. These variables might include factors such as the type of pathogen responsible for the patient’s infection, the specific type and dosage of antibiotics administered during the patient’s ICU stay, and more. As a result, further randomized controlled trials are necessary in the future to provide a more comprehensive understanding of this subject.

## Conclusion

In this study, we found that OND exposure might be associated to lower in-hospital, 28-day, and 90-day mortality rates in critically ill patients with sepsis. This study indicated that OND might help to improve the prognosis of patients with sepsis, which required further studies.

## Data availability statement

The original contributions presented in the study are included in the article/supplementary material. Further inquiries can be directed to the corresponding authors.

## Ethics statement

Ethical approval was not required for the studies involving humans because The data for this article came from the MIMIC-IV database, a public database that is available to the public in anonymous form, with our authors obtaining research consent. The studies were conducted in accordance with the local legislation and institutional requirements. Written informed consent for participation was not required from the participants or the participants’ legal guardians/next of kin in accordance with the national legislation and institutional requirements because The data for this article came from the MIMIC-IV database, a public database that is available to the public in anonymous form, with our authors obtaining research consent.

## Author contributions

BY: Conceptualization, Data curation, Formal analysis, Visualization, Writing – original draft, Writing – review & editing. YZ: Conceptualization, Data curation, Formal analysis, Investigation, Validation, Writing – original draft, Writing – review & editing. XZ: Investigation, Software, Validation, Visualization, Writing – original draft, Writing – review & editing. TL: Data curation, Formal analysis, Resources, Validation, Writing – review & editing. KN: Software, Validation, Writing – review & editing. ZW: Software, Validation, Writing – review & editing. XJ: Writing – review & editing, Validation, Investigation. XL: Supervision, Validation, Writing – review & editing. HQ: Conceptualization, Supervision, Validation, Writing – review & editing. CS: Writing – review & editing.
